# Can within-subject comparisons of thermal thresholds be used for diagnostic purposes?

**DOI:** 10.1016/j.cnp.2021.01.002

**Published:** 2021-02-04

**Authors:** Ø. Dunker, M.U. Lie, K.B. Nilsen

**Affiliations:** a Research and Communication Unit for Musculoskeletal Health (FORMI), Oslo University Hospital, Oslo, Norway; b Department of Neurology and Clinical Neurophysiology, Oslo University Hospital, Oslo, Norway; c Faculty of Medicine, University of Oslo, Oslo, Norway; d Oslo Metropolitan University, Oslo, Norway

**Keywords:** Quantitative thermal testing, Thermal thresholds, Thermal testing, Thermal perception, Normative data, Reference values

## Abstract

**Objective:**

Quantitative thermal testing (QTT) is a psychophysical assessment method of small nerve fibers that relies on reference material to assess function. Normal limits for within-subject comparisons of thermal thresholds are scarce, and their association with age, height and sex is not fully elucidated. The aim of this study was to investigate the normal limits for distal-proximal– and contralateral homologous comparisons of thermal thresholds with QTT, and their association with age, sex or height.

**Methods:**

Fifty healthy volunteers ages 20–79 participated in the experiment. Cold detection thresholds (CDT), warm detection thresholds (WDT), heat pain thresholds (HPT), and cold pain thresholds (CPT) were measured bilaterally at the thenar eminence, anterior thigh, distal medial leg and foot dorsum. Sample normal limits were calculated as (mean) ± 2 SD.

**Results:**

Forty-eight subjects were included in the analysis. CPT was excluded from all analyses due to a large floor-effect. Sample normal limits for side-differences ranged from 1.8 to 7.2 °C for CDT, 2.4–6.8 °C for WDT and 3.2–4.0 °C for HPT, depending on anatomical site. For distal-proximal comparisons, sample normal limits ranged from 4.0 to 8.7 °C for CDT, 6.0–14.0 °C for WDT and 4.2–9.0 °C for HPT, depending on the pairs compared. Age was associated with side-differences for CDT in the thenar eminences (p < 0.001) and distal medial legs (p < 0.002), and with 11 of 18 distal-proximal comparisons (p < 0.01).

**Conclusions:**

The normal limits for distal-proximal- and contralateral homologous thermal thresholds were wide, and thus of limited use in a clinical setting, although the reported values may be somewhat inflated by low sample-size and consequent age-pooling. Age, but not sex or height, was associated with contralateral differences in CDT in the thenar eminences and distal medial legs, and with most distal-proximal differences.

**Significance:**

Due to wide normal limits, we advise caution when utilizing relative comparisons of thermal thresholds for diagnostic purposes.

## Highlights

• Normal limits for within-subject comparisons of thermal thresholds are wide.
• Our findings advocate for site-specific normal values of adequate resolution.
• The difference between distal and proximal thresholds increase drastically with age.

## 1 Introduction

Small-fiber neuropathies are the result of damage to the small peripheral nerves, and typically presents as a symmetrical, length-dependent polyneuropathy that can give a wide variety of either sensory or autonomic symptoms, or a combination of both (Levine, 2018, Terkelsen et al., 2017). Most peripheral neuropathies involve both large and small fibers, but pure small-fiber neuropathies are increasingly being regarded as distinctive (Levine, 2018, Peters et al., 2013, Terkelsen et al., 2017).

Quantitative thermal testing (QTT) is an assessment method of sensory function in small, thinly myelinated (A-delta) and unmyelinated (C) nerve fibers, including their central pathways (Krumova et al., 2012). The use of QTT may help identify small-fiber involvement, when used in conjunction with clinical examination and other tests for suspected cases of small-fiber neuropathy, such as measurements of intra-epidermal nerve fiber density, laser evoked potentials (LEPS), contact heat evoked potentials (CHEPS) or nerve conduction studies. (Backonja et al., 2013, Krumova et al., 2012, Lagerburg et al., 2015, Lefaucheur et al., 2015, Løseth et al., 2006, Scherens et al., 2009, Siao and Cros, 2003). As psychophysical testing is rather complex, QTT should not be used alone for the evaluation of patients with small fiber neuropathy.

Valid reference values are an important prerequisite for QTT. Although the literature is somewhat equivocal with regards to covariates, it is advised to adjust for age (Guergova and Dufour, 2011, Hafner et al., 2015, Magerl et al., 2010, Rolke et al., 2006a, Siao and Cros, 2003), sex (Bakkers et al., 2013, Blankenburg et al., 2010, Fillingim et al., 1998, Siao and Cros, 2003, Wang et al., 2018) and possibly height (Bartlett et al., 1998, Kelly et al., 2005, Lilliesköld and Nordh, 2018, Torgén and Swerup, 2002). A wide range of distinct reference materials have been reported, e.g. for children and adolescents (Blankenburg et al., 2010, Meier et al., 2001), Hispanic Latino and African American populations (Gonzalez-Duarte et al., 2016, Powell-Roach et al., 2019), wide age-spans (Bartlett et al., 1998, Hilz et al., 1999, Pfau et al., 2014, Rolke et al., 2006a), and a number of anatomical sites (Hilz et al., 1999, Lilliesköld and Nordh, 2018, Meh and Denišlič, 1994). Great efforts have also been made to standardize experimental variables and methods of analysis (Magerl et al., 2010, Pfau et al., 2014, Rolke et al., 2006a, Rolke et al., 2006b), allowing for greater external validity. However, most published reference material shares the commonality of wide normal limits, due to large inter-individual variability, directly affecting the diagnostic sensitivity of QTT with regards to small-fiber neuropathy.

Instead of comparing thermal thresholds to absolute reference material, it is possible to perform within-subject comparisons across anatomical- or contralateral sites. Relative comparisons between distal and proximal areas are an important part of the clinical neurological examination for sensory abnormalities, including the assessment of thermal hyperesthesia and hypoalgesia. Such comparisons could be especially beneficial if they show lower variability, and thus narrower normal limits, or if they are associated with fewer covariates, allowing for more generalized reference data. Comparing thermal thresholds across distal-proximal sites could be particularly useful in instances when patients experience symmetrical pain or sensory alterations. Previous investigations have failed to find significant side-differences for QTT (Kemler et al., 2000, Lilliesköld and Nordh, 2018, Meh and Denišlič, 1994, Rolke et al., 2006a) and it has been well established that there are differences between anatomical sites, with a likely distal-proximal gradient of increasing sensitivity (Bakkers et al., 2013, Meh and Denišlič, 1994, Siao and Cros, 2003, Stevens and Choo, 1998). However, attempts to quantify normal limits for such comparisons are scarce (Kemler et al., 2000), and the association between relative comparisons and age, sex and height is largely untested. This crucial research gap should be explored further, as much of the clinical value of within-subject comparisons of thermal thresholds rests on the knowledge of normal variability in healthy individuals.

Thus, the aim of this study is to investigate the normal limits for distal-proximal– and contralateral homologous comparisons of thermal thresholds with QTT, and their association with age, sex or height.

## 2 Methods

### 2.1 Study design

An experimental, cross-sectional study was designed to compare thermal thresholds with regards to side-differences and distal-proximal gradients. Four sites were measured bilaterally: the thenar eminence; the anterior thigh, 10 cm superior to the patellar base in mid-line; the distal medial leg, directly superior and posterior to the medial malleolus, and; the foot dorsum, at the dorsal aspect of metatarsals II-III.

The protocol included cold detection threshold (CDT), warm detection threshold (WDT), heat pain threshold (HPT) and cold pain threshold (CPT). Testing order of the specific sites was randomized in advance, and a pre-test of two cold- and warm detection threshold stimuli was performed to familiarize the subjects with the procedure.

A male experimenter carried out all experiments. The QTT protocol, placement of instruments, room temperature, experimenter’s clothing, the instructions and lighting was standardized. Participants were not allowed to observe instrument readouts.

The study was approved by Regional Committees for Medical and Health Research Ethics (REC), project no. 2010/2927. All participants provided written consent, and the study was conducted in accordance with the Declaration of Helsinki. Subjects received a gift certificate of NOK 250 for participation.

### 2.2 Subjects

The required sample size for paired comparisons were calculated in advance (Rosner, 2015). For side-differences, a minimal clinical significance of 1 °C and standard deviation of 2 °C was used, while these values were 2 °C and 3 °C for distal-proximal comparisons, respectively. A minimum of 31 subjects were needed to detect a side-difference of 1 °C, with type I and II error rates of 0.05 and 0.2, respectively, and 18 were needed for distal-proximal comparisons.

Healthy men and women, ages 20–79, were recruited through advertisements at Oslo University Hospital, local universities, gyms, centers for the elderly, and on social media. Recruitment efforts focused on achieving an equivalent representation of sex and ages. Exclusion criteria were: cancer (current or previously), diabetes, radiculopathy, chronic pain (average NRS ≥ 1 for ≥ 3 months, last two years), pregnant or breastfeeding, limited capacity for consent, personal acquaintance of experimenter, or any disease of nerves, muscles, or of the brain that could influence normal nervous function, including psychiatric illnesses. Subjects were requested not to work nightshifts within 48 h of the experiment, to not consume alcohol in the last 12 h before the experiment, or consume pain-killers the same day as the experiment.

### 2.3 Test protocol

Thermal stimulus was applied with a 30 × 30 mm Peltier thermode (Medoc, Ramat Yishai, Israel), by method of limits. The thermode was held in place by the experimenter. Baseline temperature was 32 °C, with a range of 0–52 °C. The ramp-rate was 1 °C/s for all tests, and the thermode returned to baseline at 1 °C/s for detection thresholds and 5 °C/s for pain thresholds. Inter-stimulus-intervals were 4–6 s for all tests.

Subjects lay supine on a treatment table, with the back rest at approximately 120–135° incline. Pillows were used for head-support and placed under the subjects’ knees, and a duvet helped regulate skin temperature. Immediately prior to testing, the skin temperature was measured at each site with an 826-T2 hand-held infrared thermometer (Testo SE & Co., Pennsylvania, USA), held perpendicular to the skin’s surface at a standardized distance of 1 cm. A re-usable heat pack was applied where skin temperature was <30 °C for the lower extremities and <32 °C for the thenar eminence. Excessive body-hair was removed with scissors.

CDT, WDT and HPT were measured in succession for each site, followed by CPT in the distal medial legs and feet dorsa, in the same order. Absolute temperature thresholds were recorded.

Scripted, verbal instructions were used. Participants were informed of the procedure in its entirety before testing began, and reminded of the current modality before each test. For CDT and WDT, subjects were asked to press a trigger at the first sensation of cool or warmth. Similarly, for HPT and CPT, the cue was to press the trigger at the first sensation of pain, typically when the thermal stimulus begins to induce a stinging, burning or aching sensation. Subjects were advised that thermal pain thresholds are not a measure of pain tolerance. A response was considered invalid and repeated once if it deviated substantially from contemporaneous measurements, or if the subject admitted to an accidental response.

### 2.4 Data computation and analysis

Statistical analyses were performed using IBM SPSS Statistics v. 25 (Armonk, NY: IBM Corp.) P-values were regarded as significant at ≤0.05, and Bonferroni correction for multiple testing was applied. Correlation values were interpreted in accordance with Mukaka (2012): negligible correlation ± 0.0–0.3, low correlation ± 0.3–0.5, moderate correlation ± 0.5–0.7, high correlation ± 0.7–0.9 and very high correlation ± 0.9–1.0. Thermal thresholds were calculated to express absolute change from the baseline of 32 °C (Δ°C).

Data distribution was assessed in preliminary analyses by use of histograms, boxplots and Q-Q plots. The arithmetic mean of five (CDT, WDT) or three (HPT, CPT) measurements was used in the analysis. Data from each subject was excluded from the presentation of sample thresholds and for calculating regression equations if the delta value of a thermal threshold was > 3 times the arithmetic mean (detection thresholds)-, <1/3 the arithmetic mean (pain thresholds)-, or if exceeding ± 3 SD from the arithmetic mean of the remaining data points. Invalid measurements due to a test’s floor- or ceiling effect were not replaced or included in the final analysis.

Side-differences were determined by use of multiple paired t-tests. The distal-proximal gradients were examined by repeated measures ANOVA with post-hoc analysis for pairwise comparisons. Sample normal limits were calculated as mean ± 2 SD for pairwise comparisons that showed statistically significant differences, otherwise ± 2 SD.

Pearson- or Spearman correlation was used to determine the association between side-differences or distal-proximal gradients, and age, sex and height.

## 3 Results

### 3.1 Study sample

**Fig. 1:**
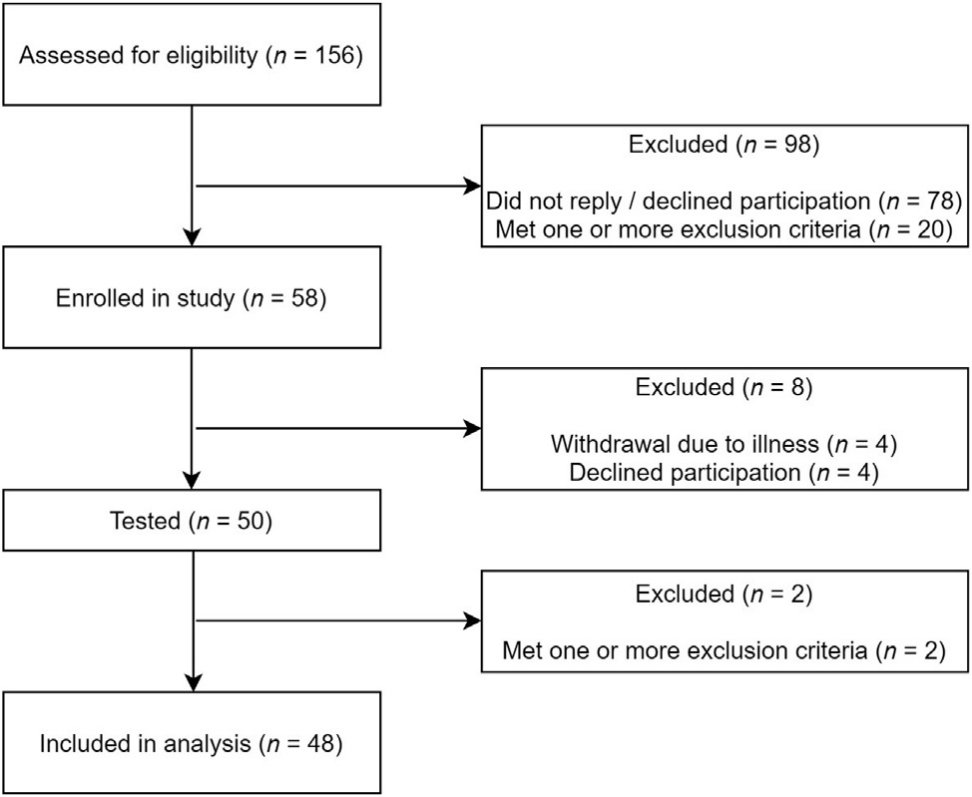
Flow chart of the study inclusion process.

Forty-eight subjects were included in the analysis. Participants were 46 (SD ± 15.6) years old, 52% of whom female. The inclusion process is displayed in Fig. 1 and sample characteristics are presented in Table 1.

**Table 1:** Sample Demographics (n = 48).

Variable	n (%/SD)
Sex, females	25 (52)

Age, years	46 (15.6)
20–29	10 (21)
30–39	9 (19)
40–49	9 (19)
50–59	10 (21)
60–69	6 (13)
70–79	4 (8)

Height, cm	174 (8.8)

BMI, kg/m^2^, mean	25 (3.9)

Relationship status	
Married/partner	28 (58)
Single	20 (42)

Education	
Primary school, 10 years	0 (0)
High school, 1–2 years	0 (0)
High school, 3 years	0 (0)
Vocational high school	5 (10)
College/university < 4 years	19 (40)
College/university ≥ 4 years	24 (50)

Current smoker, yes	3 (6)

Current snus-user, yes	5 (10)

Alcohol consumption	
Never	0 (0)
Not in > 12 months	2 (4)
<= 1 per month	12 (24)
2–4 times per month	21 (42)
2–3 times per week	11 (22)
>= 4 times per week	2 (4)

Abbreviations: SD = Standard Deviation.

Two subjects were excluded from the analysis of CDT in the distal medial leg, 34 from CPT in the foot dorsum, and 37 from CPT in the distal medial leg, due to reaching the test’s floor value of 0 °C. The large floor-effect for CPT precluded calculation of relative CPT comparisons.

Some outlying data was identified and removed for CDT in the foot dorsum (*n* = 2), distal medial leg (*n* = 5) and thenar eminence (*n* = 2); for WDT in anterior thigh (*n* = 2) and thenar eminence (*n* = 1); and for HPT in anterior thigh (*n* = 1) and thenar eminence (*n* = 1). Sample thermal thresholds are listed in Table 2.

**Table 2:** Sample thermal thresholds (Δ from 32 °C).

	Test site	Mean (SD)
CDT	Foot dorsum^b^	4.1 (2.8)
	Distal medial leg^c^	4.0 (2.0)
	Anterior thigh	3.1 (1.7)
	Thenar eminence^b^	1.5 (0.8)

WDT	Foot dorsum	7.6 (3.8)
	Distal medial leg	9.8 (3.4)
	Anterior thigh^b^	4.3 (1.5)
	Thenar eminence^a^	2.3 (1.0)

HPT	Foot dorsum	13.5 (3.3)
	Distal medial leg	14.8 (2.5)
	Anterior thigh^a^	13.8 (2.7)
	Thenar eminence^a^	12.6 (3.5)

Abbreviations: SD = Standard Deviation; CDT = Cold Detection Threshold; WDT = Warm Detection Threshold; HPT = Heat Pain Threshold.

a n = 47.

b n = 46.

c n = 43.

### 3.2 Distal-proximal gradients for quantitative thermal testing

**Fig. 2:**
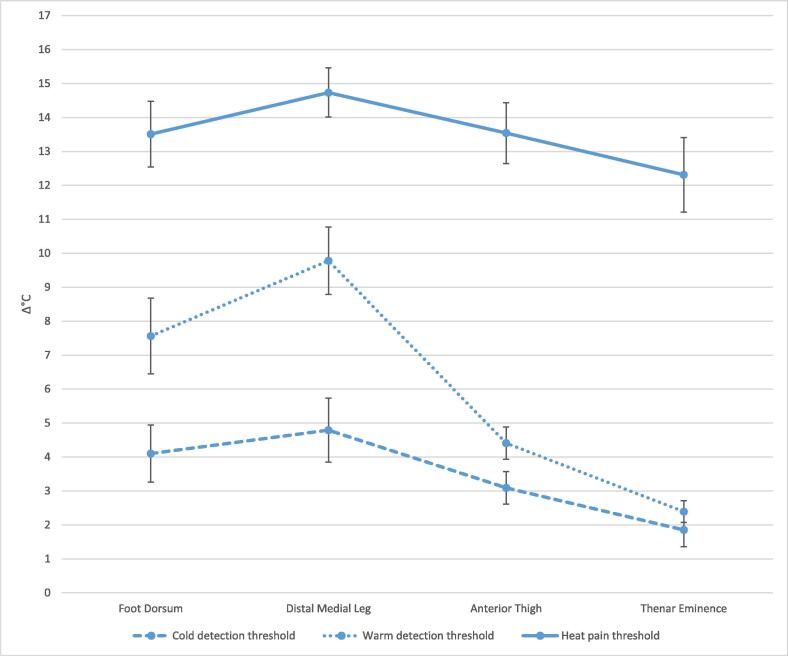
Thermal thresholds (change from baseline °32C) for cold detection, warm detection and heat pain, in the foot dorsum, distal medial leg, anterior thigh and thenar eminence. Error bars represent 95% confidence intervals.

Repeated measures ANOVA with Greenhouse-Geisser correction detected differences in means when comparing CDT, WDT and HPT (p < 0.001). Post-hoc analysis with Bonferroni correction for multiple testing displayed a general distal-proximal gradient for CDT, WDT and HPT (Table 3). The distal-proximal gradient’s linearity was violated by the distal medial leg being less sensitive than the foot dorsum for WDT (p < 0.001) and HPT (p < 0.001), with no difference detected for CDT (p = 0.170) (Fig. 2). In addition, no significant difference was found between the foot dorsum and anterior thigh (p = 0.932) or thenar eminence (p = 0.016) for HPT. Sample normal limits for distal-proximal comparisons ranged from 4.0 to 8.7 °C for CDT, 6.0–14.0 °C for WDT and 4.2–9.0 °C for HPT.

**Table 3:** Distal-proximal gradients for thermal thresholds (Δ°C).

	Pairwise comparisons				Mean difference (SD)	P-value	Sample normal limits
		Mean change from baseline (32 °C)					
**CDT**^d^	Foot dorsum	3.9	4.5	Distal medial leg	−0.6 (2.7)	0.170	5.4^c^
			3.0	Anterior thigh	0.9 (2.0)	0.002^a^	4.0^c^
			1.8	Thenar eminence	2.1 (2.5)	<0.001^b^	7.1
	Distal medial leg	4.5	3.0	Anterior thigh	1.5 (2.8)	0.001^b^	7.1
			1.8	Thenar eminence	2.7 (3.0)	<0.001^b^	8.7
	Anterior thigh	3.0	1.8	Thenar eminence	1.2 (1.6)	<0.001^b^	4.4

**WDT**	Foot dorsum	7.6	9.8	Distal medial leg	−2.2 (2.5)	<0.001^b^	7.2
			4.4	Anterior thigh	3.2 (3.2)	<0.001^b^	9.6
			2.4	Thenar eminence	5.2 (3.4)	<0.001^b^	12.0
	Distal medial leg	9.8	4.4	Anterior thigh	5.4 (2.9)	<0.001^b^	11.2
			2.4	Thenar eminence	7.4 (3.3)	<0.001^b^	14.0
	Anterior thigh	4.4	2.4	Thenar eminence	2.0 (2.0)	<0.001^b^	6.0

**HPT**	Foot dorsum	13.5	14.7	Distal medial leg	−1.2 (1.5)	<0.001^b^	4.2
			13.5	Anterior thigh	0.0 (2.1)	0.932	4.2^c^
			12.3	Thenar eminence	1.2 (3.4)	0.016	6.8^c^
	Distal medial leg	14.7	13.5	Anterior thigh	1.2 (2.0)	<0.001^b^	5.2
			12.3	Thenar eminence	2.4 (3.3)	<0.001^b^	9.0
	Anterior thigh	13.5	12.3	Thenar eminence	1.2 (2.2)	<0.001^b^	5.6

Abbreviations: CDT = Cold Detection Threshold, WDT = Warm Detection Threshold, HPT = Warm Detection Threshold, SD = Standard Deviation, CI = Confidence Interval.

Δ°C = Change from baseline 32 °C.

Sample normal limit = Mean ± 2 SD.

^c^Sample normal limit = 2 SD.

^a,b^Significant at p ≤ 0.05 and 0.01, respectively, after Bonferroni adjustments for multiple testing.

d n = 46.

### 3.3 Side-differences for quantitative thermal testing

Side-differences for thermal thresholds presented in Table 4. A difference of −1.4 °C (−2.1 °C to −0.6 °C) was found for WDT in the feet dorsa (p = 0.001), significant at p < 0.05 after Bonferroni correction for multiple testing. Sample normal limits for side-differences ranged from 1.8 to 7.2 °C for CDT, 2.4–6.8 °C for WDT and 3.2–4.0 °C for HPT.

**Table 4:** Side-differences for thermal thresholds (Δ°C).

	Test site	Right Side (mean, SD)	Left side (mean, SD)	Side-difference (SD)	P-value	95% CI	Sample normal limits
**CDT**	Thenar eminence	0.9 (1.9)	0.8 (1.6)	0.2 (0.9)	0.254	−0.4 to 0.1	1.8
	Anterior thigh	2.9 (1.7)	3.3 (1.9)	0.4 (1.4)	0.090	−0.1 to 0.8	2.8
	Distal medial leg^c^	4.5 (3.5)	4.7 (3.4)	0.2 (3.6)	0.647	−0.8 to 1.3	7.2
	Foot dorsum	4.0 (3.0)	4.2 (3.0)	0.2 (2.1)	0.543	−0.4 to 0.8	4.2

**WDT**	Thenar eminence	2.7 (1.5)	2.1 (0.9)	0.5 (1.2)	0.004	0.2 to 0.8	2.4
	Anterior thigh	4.4 (2.0)	4.4 (2.0)	0.0 (2.3)	0.978	−0.7 to 0.7	4.6
	Distal medial leg	9.4 (3.8)	10.1 (3.5)	0.7 (2.7)	0.083	−1.5 to 0.1	5.4
	Foot dorsum	6.9 (4.0)	8.2 (4.1)	1.4 (2.7)	0.001^a^	−2.1 to −0.6	6.8^b^

**HPT**	Thenar eminence	12.2 (4.1)	12.5 (3.7)	0.3 (2.0)	0.324	−0.9 to 0.3	4.0
	Anterior thigh	13.6 (3.3)	13.5 (3.1)	0.0 (2.0)	0.891	−0.5 to 0.6	4.0
	Distal medial leg	14.7 (2.5)	14.8 (2.7)	0.0 (1.6)	0.873	−0.5 to 0.4	3.2
	Foot dorsum	13.1 (3.6)	13.9 (3.3)	0.8 (1.9)	0.005	−1.4 to −0.3	3.8

Abbreviations: CDT = Cold Detection Threshold, WDT = Warm Detection Threshold, HPT = Warm Detection Threshold, SD = Standard Deviation, CI = Confidence Interval.

Δ°C = Change from baseline 32 °C.

Sample normal limit = 2 SD.

a Significant at p < 0.05 after Bonferroni adjustment for multiple testing.

b Mean + 2 SD.

c n = 46.

### 3.4 Relation to age, sex and height

A low correlation was found between age and side-differences for CDT between the thenar eminences (r = 0.46, p = 0.001) and distal medial legs (r = 0.44, p = 0.002). Age was moderately correlated with the CDT gradient between the foot dorsum and anterior thigh (r = 0.52, p < 0.001), the foot dorsum and thenar eminence (r = 0.59, p < 0.001) and between the distal medial leg and thenar eminence (r = 0.52, p < 0.001); with the WDT gradient between the foot dorsum and anterior thigh (ρ = 0.65), the foot dorsum and thenar eminence (r = 0.65, p < 0.001), the distal medial leg and anterior thigh (r = 0.52, p < 0.001) and between the distal medial leg and thenar eminence (r = 0.50, p < 0.001); and with the HPT gradient between the foot dorsum and anterior thigh (r = 0.53, p < 0.001), the distal medial leg and thenar eminence (r = 0.59, p < 0.001) and between the anterior thigh and thenar eminence (r = 0.56, p < 0.001). A high correlation between age and thermal threshold gradient was found for HPT between the foot dorsum and thenar eminence (r = 0.72, p < 0.001).

**Fig. 3:**
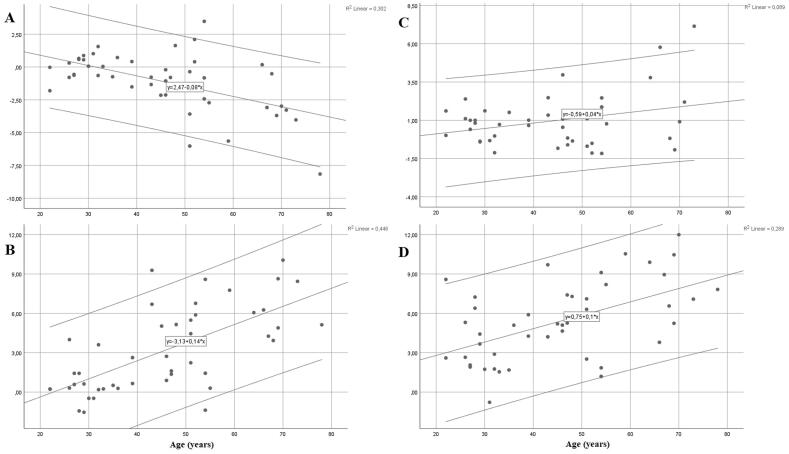
Scatterplot with fitted line and 95% prediction intervals for difference in (A) cold detection thresholds between foot dorsum and anterior thigh; (B) warm detection thresholds between foot dorsum and anterior thigh; (C) cold detection thresholds between distal medial leg and anterior thigh; and (D) warm detection thresholds between distal medial leg and anterior thigh. Note that C shows an inverse trend of cold detection thresholds decreasing with age (p = 0.052), possibly due to somewhat outlying data.

The correlation between age and relative comparisons of the feet dorsa or distal medial legs and anterior thigh for CDT and WDT are presented in Fig. 3.

No significant correlations were found for height or sex.

## 4 Discussion

We found significant differences for most distal-proximal comparisons of thermal thresholds, while side-differences were practically non-existent. The large inter-subject variability in our sample resulted in wide limits of normality for most relative comparisons. Age was correlated with two of the contralateral- and most of the distal-proximal differences, while no correlation was found between sex or height and side-differences or distal-proximal gradient, suggesting that the latter covariates need not be accounted for when establishing normal limits for within-subject comparisons.

### 4.1 Distal-proximal comparisons

A general distal-proximal gradient of increasing thermal sensitivity, with differences as high as 100-fold between the face and feet, has previously been reported (Bakkers et al., 2013, Blankenburg et al., 2010, Claus et al., 1987, Kelly et al., 2005, Lilliesköld and Nordh, 2018, Meh and Denišlič, 1994, Stevens and Choo, 1998, Yarnitsky and Sprecher, 1994). Although a few comparisons revealed no differences between anatomical sites, i.e. CDT foot dorsum-distal medial leg, HPT foot dorsum-anterior thigh and HPT foot dorsum-thenar eminence, our findings confirm the presence of a general distal-proximal gradient of increasing thermal sensitivity. However, when zoomed in on the distal lower extremities, the data aligns with a previous report (Zhang et al., 2017) in showing that the gradient is non-linear. In the present study, the distal medial calf exhibited equal sensitivity to the foot dorsum for CDT, and was less sensitive for WDT and HPT, while the large floor effect deterred any conclusion regarding CPT. The difference between the foot dorsum and distal medial leg may be due to differences in nerve-fiber density or phenotype distribution, e.g. C/Aδ low threshold mechanoreceptors or C-mechanocold (Lawson, 2002, Paricio-Montesinos et al., 2020). It could also be affected by varying cortical representation, as for instance the big-toe, heel and hip have all been shown to have larger representations and peak activations of the Brodmann areas (BA 3b, 1, 2) than the calf (Akselrod et al., 2017), although the difference is unlikely to be as pronounced between the foot dorsum and distal medial leg for thermal sensitivity.

The finding does have clinical implications, as for instance Maier et al. (2010) reports tentatively using absolute reference values for the hand and feet in the upper and lower body, respectively. According to our findings, e.g. utilizing reference values for the feet dorsa to assess small-fiber function in the relatively adjacent distal medial legs, could result in an increase in false positives for WDT hypoesthesia or false negatives for HPT hyperalgesia. This may indicate that the foot dorsum is a more sensitive test site than the distal medial leg, but the choice of test-site will also depend on availability of local reference values and the indication for the test. Consequently, we surmise that adequate care should be taken to ensure sufficiently high resolution of anatomical sites when creating absolute reference material or determining normal limits for inter-region comparisons, and we advise that only site-specific reference values are used clinically until the required resolution is fully elucidated.

It is uncertain whether inter-subject variability of thermal thresholds increase with distality, as findings from previous research are equivocal (Bartlett et al., 1998, Dyck and Karnes, 1993, Lilliesköld and Nordh, 2018, Meh and Denišlič, 1994, Moravcová et al., 2005, Rolke et al., 2006b, Zhang et al., 2017). However, we show that this may be true for inter-region comparisons. This could mean that comparisons across anatomical sites are less useful when they involve the feet dorsa or distal medial legs, which, regrettably, are high-prevalence areas for distal neuropathies.

Rolke et al. (2006a) reported that region, e.g. face *vs.* foot had a larger effect on thermal thresholds than age or sex, but found no increase in sensitivity by comparing regions instead of using absolute reference data. It cannot be ruled out that the findings of Rolke et al. (2006a) are due to quite distal comparisons, i.e. that adjacency of the anatomical sites compared may influence diagnostic sensitivity somehow; yet our findings of wide normal limits for neighboring anatomical sites raises doubts that this is the case. Indeed, comparisons of detection thresholds between adjacent sites in the lower extremities show normal limits of 5.4–11.2 °C in our sample. Still, it must be noted that the aged part of our age-pooled sample may inflate these limits somewhat due to higher variability, and thus, larger samples that allow for age stratification are needed in future research to more accurately explore distal-proximal comparisons across age-groups.

### 4.2 Side-differences

Previous investigations have reported some gains in diagnostic sensitivity when utilizing contralateral comparisons, either on its own, or as a supplement when the patient’s results are within normal ranges of absolute reference values (Blankenburg et al., 2010, Maier et al., 2010, Pfau et al., 2014, Rolke et al., 2006a). However, in line with what our results may imply, these findings mainly concern thermal pain thresholds, with sensitivity gains for detection thresholds often being negligible, thus possibly diminishing the clinical value of the findings. Still, since sensitivity and specificity are inversely, proportionally related, the wide normal limits may increase the test’s specificity, potentially allowing for a confirmatory role in a cluster of tests assessing small-fiber function.

Although the present study cannot rule out that a side-difference may exist for WDT in the feet dorsa, we suspect our singular finding to be due to an unknown systematic error. Previous investigations have not found such a side-difference (Kemler et al., 2000, Lilliesköld and Nordh, 2018, Meh and Denišlič, 1994, Rolke et al., 2006a), and even though we applied a conservative method to correct for multiple testing, a false positive could be caused by experimental errors. A possible explanation is the use of a relatively small thermode (30x30mm), and subsequent low spatial resolution on the uneven surface of the foot dorsum. Through a systematic difference in how the thermode was manually applied by the experimenter, small areas with a lower warmth receptor density could theoretically be targeted, or spatial summation could be influenced by varying degrees of contact with the skin (Backonja et al., 2013, Bakkers et al., 2013, Gelber et al., 1995).

The normal limits for side-differences presented are somewhat comparable to previous findings, but due to the large impact of methodology, anatomical sites measured and statistical decisions made, heterogeneity prohibits simple comparisons between studies. Rolke et al. (2006a) investigated side-differences of 180 subjects with the DFNS protocol, but measured the cheeks, hands dorsa and feet dorsa, and pooled all data to only compare means between sides. The authors conclude that relative comparisons may be worthwhile when investigating HPT or CPT, yet a direct comparison of thermal thresholds with the present study would not be valid due to differences in test-sites and method of analysis. In another study assessing side differences in thermal thresholds, Kemler et al. (2000) reported normal limits for volar wrists and feet dorsa in 50 healthy subjects. The normal limits for side-differences for CDT, WDT and HPT in the feet dorsa were lower than in the present study, at 1.5 °C, 3.8 °C and 2.2 °C, respectively. However, this comparison may also be unreasonable, as these lower thresholds may be explained by differences in methodology, particularly the use of method of levels, the fact that a side-difference for WDT was included in our calculations, and that the relatively low sample-sizes in both studies may affect the precision.

In line with Yarnitsky and Sprecher (1994), we found that the inter-individual variability is dependent on body-site, underlining the importance of sufficiently anatomically specific reference values. In fact, the normal limits for contralateral comparisons can vary by a factor of four, exemplified by our sample normal limits for CDT of 1.8 °C for the thenar eminences and 7.2 °C for the distal medial calves. Following this, regarding a set difference between contralateral homologous sites of e.g. ≥±1°C for thermal detection thresholds or ≥±2°C for thermal pain thresholds as pathological, like Leffler and Hansson (2008), may not only be erroneous because of the narrow limit proposed, but also because different limits should systematically be applied depending on the body site examined.

An important limitation of comparing sides is that the contralateral site must be normal, limiting its use in e.g. symmetrical distal polyneuropathies. Besides, both pain and functional alterations can spread with time, i.e. enlarging the affected area or mirroring the pathology (Jancalek, 2011), for instance in complex regional pain syndrome (Maleki et al., 2000, Rasmussen et al., 2018, Veldman and Goris, 1996) or post-herpetic neuralgia (Oaklander et al., 1998). In such cases, it would be more appropriate to compare thermal thresholds across anatomical sites.

### 4.3 Relation between relative comparisons and age, sex and height

The finding of no relation between sex and side-differences is in line with previous work by Kemler et al. (2000). If sex and height is in fact inconsequential to the external validity of relative reference values, large sample sizes may be more accessible to future relative reference material, which could in turn allow for appropriate age stratification.

In some contrast to Kemler et al. (2000), we found that age was significantly correlated with side-differences for CDT in the thenar eminences and distal medial legs. Furthermore, age was associated with most of the distal-proximal comparisons, with larger differences being normal in the older patient. The effect of age on distal-proximal comparisons may be due to faster ageing of the peripheral nerves, for instance age-related perfusion impairment near the peripheral receptors, age-related reduction in inter-epidermal nerve fiber density, distal axonopathy, or age-related changes in the synthesis, transport and action of key neurotransmitters leading to higher firing thresholds (Chakour et al., 1996, Guergova and Dufour, 2011, Stevens and Choo, 1998). Accordingly, it seems necessary to adjust for age when performing relative comparisons of thermal thresholds, particularly when assessing the most distal sites. However, it should be noted that age only partly explains the variance seen in thermal thresholds in our sample, suggesting that other covariates than age, sex or height may play a role in relative comparisons of thermal thresholds.

## 5 Strengths and limitations

The external validity of our findings is strengthened by our close adherence to the DNFS protocol.

An important limitation is the relatively low sample-size, and consequent loss of possibility to stratify by age. Collecting reference material stratified for all age groups constitutes a trade-off between the amount theoretically needed and what is practically possible to achieve. We managed to include 48 persons. Partitioning this sample into small age-groups, e.g. decades would decrease the precision of the normal limits, and so the sample was pooled. Still, older individuals are likely to show high inter-individual variability, which may have increased the normal limits in our study due to a sample consisting of individuals of all ages.

We defined healthy as someone with no evidence of disease, and not evidence of no disease (Jabre, 2018). This entails that while we performed a screening interview and carefully handled abnormal tests and outliers, subclinical neuropathies may still theoretically have been present in some subjects included in the final analysis. However, we believe that our definition represents an adequate compromise between said risk and invasive testing of healthy volunteers.

With a psychophysical design consisting of 116 recorded measurements per subject, some outliers are to be expected. However, we believe that our careful effort of blunting single-test-outliers by utilizing the arithmetic mean for each test site and modality, only removing relatively extreme outliers (mostly delta-values, preserving a mean value also in these subjects), and not replacing invalid measurements that are only considered invalid due to a somewhat arbitrarily limited test range (0–52 °C), lets our data represent much of the true variation seen in- and between healthy subjects.

Each trial lasted about one hour without breaks; it is thus possible that attention and reaction-time was diminished through the last tests, and since the test-order was randomized, this could theoretically lead to a global increase in inter-subject variability and widening of the sample normal limits. However, failure to randomize could lead to higher thresholds in only the last measurements, creating a systematic bias, and thus we believe that this source of error is best managed globally.

## 6 Conclusion

The normal limits for distal-proximal- and contralateral homologous thermal thresholds were wide, and thus of limited use in a clinical setting. Age, but not sex or height, was associated with most distal-proximal differences, and with contralateral differences in cold detection thresholds in the thenar eminences and distal medial legs. In addition, our data confirms that the distal medial legs may be equally– or less sensitive than the feet dorsa for thermal stimuli, resulting in a non-linear distal-proximal gradient, and highlighting the need for site-specific reference values for thermal thresholds in general.

## Funding

Funding was received from Oslo University Hospital and Oslo Metropolitan University. Neither sponsor was involved in design, data collection, analysis, interpretation, writing of the report or in the decision to submit the article for publication.

## Declaration of interest

None.

## 9 Contributors

All authors contributed to conception and design, while Ø. Dunker performed acquisition- and analysis of data. Ø. Dunker drafted the article, while K.B. Nilsen and M.U. Lie made critical revisions to the text. All authors approve of the final version.
